# 

**Published:** 1873-04-15

**Authors:** 


Illinios State Medical Society.—The next annual meeting of our State Society will be held on Tuesday, May 20th, 1873, in Bloomington, Illinois, commencing at 10 o'clock, A. M. The location is a pleasant one, and we hope the profession in every part of the State will be represented.

